# Patterns and Drivers of Aboveground Insect Diversity along Ecological Transect in Temperate Grazed Steppes of Eastern Eurasian

**DOI:** 10.3390/insects14020191

**Published:** 2023-02-15

**Authors:** Xiaoxiao Song, Lei Ji, Guangming Liu, Xiao Zhang, Xiangyang Hou, Shujing Gao, Ning Wang

**Affiliations:** 1 Key Laboratory of Biohazard Monitoring and Green Prevention and Control in Artificial Grassland, Ministry of Agriculture and Rural Affairs, Institute of Grassland Research, Chinese Academy of Agricultural Sciences, Hohhot 010010, China; 2 Shandong Engineering Research Center for Environment-Friendly Agricultural Pest Management, College of Plant Health and Medicine, Qingdao Agricultural University, Qingdao 266109, China

**Keywords:** climate factors, grazing intensity, insect communities, plant diversity, steppe type, transect

## Abstract

**Simple Summary:**

Understanding the macro pattern and mechanisms of variation in species diversity along environmental gradients is one of the most important objectives in ecology. However, there have been few large-scale studies on the specific mechanisms by which climate and human activities affect insect diversity along the ecological transect. This study revealed the diversity patterns of insect communities along the ecological transect in the Eastern Eurasian Temperate Steppe, investigated the effects of environmental factors on its diversity in two types of steppes, and assessed the influence of plant diversity alterations on these effects. Our results revealed a breakpoint of insect diversity that divided typical and desert steppe communities. Climate factors and grazing intensities combine to influence insect diversity along the transect, and these effects are mediated through plant diversity. These findings provide a reference for the diversity patterns of insects along ecological gradients in the Eastern Eurasian Temperate Steppe and different conservation strategies of biodiversity in typical and desert steppes.

**Abstract:**

Insects are important components of biodiversity and play significant roles in the steppe ecosystem. They are abundant, easy to sample, and sensitive to changing conditions, making them useful indicators of environmental changes. This study aims to describe patterns (α and β) of insect diversity across two steppe types (a typical steppe and a desert steppe) along the Eastern Eurasian Steppe Transect (EEST), as well as evaluate the effects of environmental variables in determining these patterns and the influence of plant diversity alterations on these effects. To this end, we collected 5244 individual insects and found an n-shaped diversity distribution along the latitudinal gradient and a significant difference in insect communities across the two steppe types. Further, the Mantel test and path analysis indicate that climate and grazing activities combine to influence insect diversity, and these effects are mediated through plant diversity, strongly supporting the role of bottom-up effects in situations of climatic and grazing pattern changes. Moreover, the contribution of plant diversity varied with steppe types and insect functional groups, with greater effects seen in the typical steppe and herbivorous insects. This indicated the importance of protecting species diversity in steppes through managing plant diversity and assessments of local environmental factors such as grazing intensity and temperature.

## 1. Introduction

The Eurasian Temperate Steppe is the largest continuously distributed steppe in the world and plays an important role in the global grassland ecosystem [1,2]. It is highly sensitive and vulnerable to climate change and is also one of the most ecologically degraded areas on earth due to the effects of human activities [3]. Unfortunately, the Eurasian Steppe is degrading rapidly due to climate change and human activities that are changing it into barren and desert land [4], with serious effects on biodiversity. Understanding the ecological potential of the Eurasian Temperate Steppe is extremely critical to have an overview of the conservation of the global steppe ecosystem [3]; this requires urgent research on the taxonomic groups that can serve as study models or bioindicators.

Insects are important components of grassland ecosystems and play significant roles in the soil biogeochemical cycle and food chain network [5]. They have been widely used to monitor environmental changes in large-scale ecosystems [6,7], as they are abundant, easy to sample, and sensitive to changing conditions; this makes them also ideal for studying distribution patterns and community differences between different habitat types. Furthermore, different insect functional groups often respond differently to environmental factors [8,9], which contributes to a comprehensive understanding of changes in the community structure. Therefore, investigating diversity patterns and the key factors affecting the overall and major functional group diversity of insects across different steppe types can lay the foundation for the community structure and ecosystem function of the steppe ecosystem.

Climate changes and human activities are key factors driving insect community dynamics. Temperature is the most important abiotic factor for insects in climate, which directly affects the growth, reproduction, and development of insects [10]. Brehm et al. [11] and Axmacher et al. [12] showed that environmental factors such as temperature and humidity significantly affected the numbers and compositions of insect species, with many insect species in the northern hemisphere expanding northwards under the influence of climate change. Precipitation plays a more significant role in arid and semi-arid ecosystems [13]. It directly affects plant community composition and above biomass, leading to changes in insect diversity [14]. Moreover, climate change also has indirect effects on the insects’ host plants, competitors, and natural enemies [15]. Grasslands are extensively used for livestock grazing, which significantly alters plant diversity [16], heterogeneity [17], and community structures [18] and, consequently, results in insect species turnover due to preferences for either shady or open habitats, potentially affecting insect populations and community dynamics [19,20,21]. All these studies indicate that plants play an important guiding role in insect species diversity. Plant species richness is often used as a proxy for food resource diversity for insects, and a positive relationship between plant diversity and insect diversity has been found [22,23]. However, most of these studies are limited to the experimental platform of a single steppe without addressing the large-scale issues of ecosystem management.

Ecological transects are an effective way to understand the current relationship between global climate change and terrestrial ecosystems and can thus suggest future trends [3]. The Eastern Eurasian Steppe Transect (EEST) (108–115° E, 39–59° N) is the first transect, which is an international transect across regions with middle and high latitudes in the Eastern Eurasian Steppe [3]. The transect has a clear thermal gradient due to the alternate effects of the East Asian monsoon and the northern cold snap and has a continuous distribution of similar vegetation, with grazing gradients across different countries and human and livestock management practices. Accordingly, it is of great importance to assess the response of key species and biological groups to global change and different intensities of human disturbance along the transect; a primary goal of using bioindicators is to reduce the complexity of environmental change down to empirically derived units of information which can improve our knowledge of the environmental change and inform future environmental management and conservation efforts. Such a study can provide valuable scientific and practical guidance for the conservation of biodiversity and sustainable grassland management.

This study examined the influence of both climate and grazing on insect diversity across two types of grasslands along the EEST region and investigated whether these effects were mediated by alterations in plant diversity. Specifically, the following questions were addressed: (a) how do climate and grazing affect plant and insect diversities in different steppe types?; (b) are the effects on insect diversity mediated directly or indirectly by alterations in plant communities resulting from interactions between different environmental variables?; and (c) do the effects of plant changes on insect diversity vary by species and steppe type?

## 2. Materials and Methods

### 2.1. Study Area

**Figure 1:**
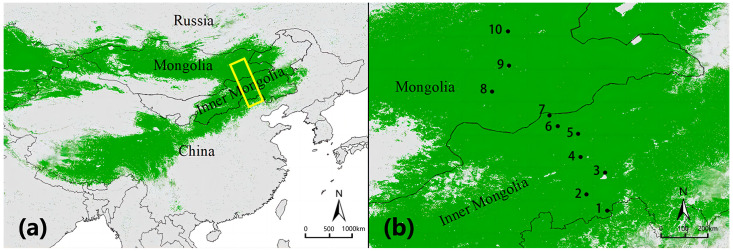
(**a**) Study area. Green indicates the distribution of the Eurasian Steppe. The yellow transect is the study area, corresponding to the Eastern Eurasian Steppe Transect (EEST) region. (**b**) Field sampling sites (black circles). Map is displayed on a WGS 1984 World Mercator projection (datum: WGS 1984).

Ten sites along the EEST region were used for investigation. This transect runs from the Great Wall of China (south) through Mongolia to Lake Baikal (north) in Russia (41.55–52.63° N, 105.53–117.17° E, 480–1647 m a.s.l.) and is 1400 km in length from north to south and 200 km wide from east to west (Figure 1). The ten sites were situated from south to north along the EEST region stretching from 42.18 to 47.65° N and from 114.18 to 116.74° E. There were seven study sites situated in the Inner Mongolian steppe subregion and three in the Mongolian steppe subregion along the latitudinal gradient ranging from typical steppe in the south to desert steppe in the north (Figure 1). A desert steppe is characterized by a cold, semi-arid continental monsoonal climate, with a mean temperature (MAT) of −1.5 °C and mean annual precipitation (MAP) of 200 mm [24]. Vegetation is mainly represented by drought-tolerant species, such as *Stipa klemenzii* and *Stipa gobica*, with low productivity and species richness. A typical steppe is characterized by a typical arid and semi-arid temperate continental climate with a MAT of 3.1 °C and MAP of 350 mm, of which approximately 80% occurs during the May–August growing season [25]. To reflect possible with-ecosystem variability, we selected two sampling sections; the sites at the south side (Sites 1, 2, and 3) were dominated by several species of the genus of *Leymus*, principally *L. chinensis*, and the sites at the north (Sites 4 and 5) were dominated by *Stipa grandis* and *Stipa krylovii*, both showing intermediate productivity and low species richness. Each site in the typical steppe was distributed in an area of about 3 km^2^, and each site in the desert steppe was distributed in an area of about 2 km^2^; the desert was about 80 km from the typical steppe. Thus, there is no risk that some sampling points would trap species of adjacent habitats, and we avoided the effect of edge. The features of the sites are summarized in Table 1 and Table S1.

### 2.2. Insect Sampling and Identification

At each sampling site, we surveyed insects using four randomly located 20 × 20 m^2^ quadrats (separated by at least 60 m from each other); within each site, there were 40 quadrats in total. To avoid temporal effects, field sampling was conducted between late July and mid-August 2012 when the grassland community biomass was highest. Insects were sampled between 09:00 and 12:00 on sunny days using a checkerboard sweep net method (38 cm in diameter), whereby samples were collected by making a total of 250 sweeps, with 5 vertical sweeps (every 5 m) and 5 horizontal sweeps (every 80 cm) [26]. The captured insects were placed in containers with 95% ethanol. Where possible, specimens were identified at the species level, with the exception of larvae due to difficulties in identification. If unable to be identified, the specimens were classified as morphospecies according to appropriate identification keys [27]. All specimens were identified in consultation with taxonomic experts and were deposited in the Entomological Museum of the Institute of Grassland Research, Chinese Academy of Agricultural Sciences in Hohhot, Inner Mongolia, China (No. IGR800075-805318).

### 2.3. Vegetation Measurement

Vegetation sampling was put down from late July to mid-August 2012. Areas of 10 × 10 m were set out at each site, with four 1 × 1 m quadrats placed randomly within the area. Within a 1 m^2^ quadrat frame, we measured four attributes: plant height (PH, average, cm), cover (PC, % of soil covered by plants), dry biomass (PB, g/m^2^), and plant Shannon Wiener index (expressed as plant diversity, PS). Plants were identified to the species or morphospecies level using specialized literature [28], and the numbers were recorded.

### 2.4. Environmental Variables

To investigate the effects of climatic variables on species abundance, the MAT and MAP values obtained at a 30 arc-second (~1 km^2^) resolution were acquired from the WorldClim database (https://www.worldclim.org, accessed on 9 June 2022). The principal human activity in the study region is grazing, and sheep densities were used as indicators of grazing intensity, which was defined as sheep ha^−1^ (GI) and was included in the environmental variables. Data on sheep densities in 2012 were obtained from the Food and Agriculture Organization of the United Nations (https://data.apps.fao.org/, accessed on 10 June 2022) and were extracted according to the locations of the different sites (Table S1). At each site, spatial geographical coordinates and elevations were recorded using a handheld GPS (eTrex Venture, Garmin, Olathe, KS, USA). The pairwise geographic distance (GEO) between sites was calculated using the “geosphere” package in R v.4.1.3 [29] according to the GPS coordinates of each site.

### 2.5. Data Analyses

R version 4.1.3 [29] was used for all statistical analyses. We used the abundance data (individual-based abundance data) by performing rarefaction and extrapolation curves to estimate sampling sufficiency in all sites (Figure S1). Sample coverage was determined using Hill’s numbers of q = 0 (i.e., presence–absence data), doubling the sizes of the reference samples [30], and using 100 bootstrap replications for the determination of confidence intervals; these were performed using the R package “iNEXT” [31]. To compare species richness, species diversity, and abundances of all insects in each site along the latitudinal gradient and explore the community pattern at the species level, we used the nonparametric Wilcoxon tests based on data from sampling sites. The variation in insect species richness and abundance with latitude were evaluated using generalized additive models (GAMs), using the “gam” function in the “mgcv” package, with the data family set to Gaussian type and the basis dimension of the smoothing function (k) to four to avoid over-parameterization. The data for the complete dataset and the typical and desert steppes were analyzed separately. Principal coordinate analysis (PCoA) with Bray–Curtis distances was used to assess the β-diversity patterns in the different steppe types. Permutational multivariate analysis of variance (PERMANOVA) was used to examine differences in communities using the “vegan” package [32] with 999 permutations.

Two approaches were used for exploring the influence of environmental variables on insect diversities. The Mantel test [33] in the “vegan” R package was used initially to examine the influence of environmental factors and geographic distance on insect community similarity between different steppe types. Seven environmental variables (MAT, MAP, GI, PH, PC, PB, and PS), their synergistic effect (ENV), and geographic distance (GEO) were used. The standardized Mantel’s r (ranging between 0 and 1) represented the strength of the association, with higher r values indicating stronger relationships, and significance was assessed using *p*-values calculated from 999 randomizations [34]. This was followed by a path analysis using three latent variables, i.e., MAT, MAP, and GI, to examine their influence on insect abundance and richness, together with the degree to which this influence was mediated by changes in vegetation [35,36]. For the inner model matrix of the path model, we hypothesized that MAT, MAP, and GI predicted species abundance and richness either directly or indirectly through their combined influence on vegetation change. MAT, MAP, and GI were used as fixed factors, while the cover, height, biomass, and Shannon–Wiener indices of the plants, as well as the insects, were used as independent variables. The partial least squares (PLS) approach [37] in the R package “plspm” was used for the path analysis. 

Redundancy analysis (RDA) using the “rda” package was used to visualize the associations between the abundance of insect orders and the vegetation. Insect order abundance in relation to the environmental variables of plant species, cover, biomass, and Shannon–Wiener index was used to compile ordination plots using the “envfit” function of the “vegan” package with 999 permutations; variables with *p* < 0.01 were selected as independent variables. The associations between the plant Shannon–Wiener indices, the total insect abundance, and the relative abundances of six dominant insect species were analyzed using linear regression with the “lm” function in the package “lme4” [38]. 

## 3. Results

### 3.1. Species α- and β-Diversity

**Figure 2:**
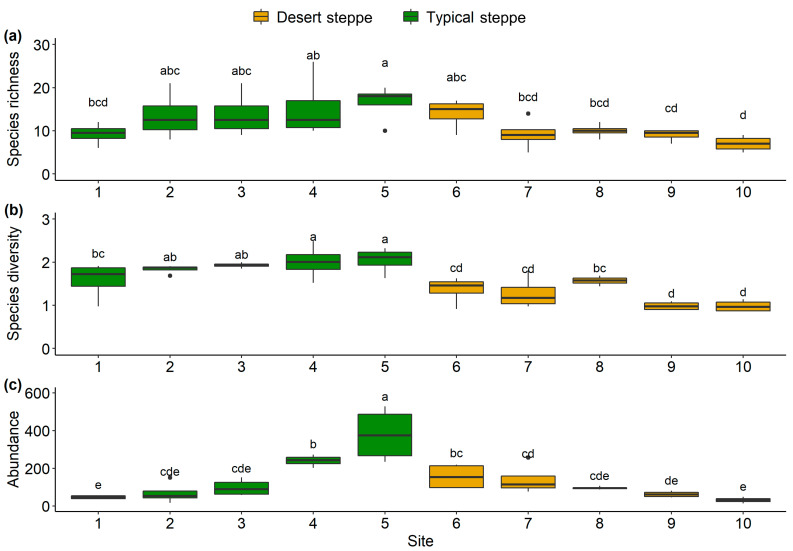
Species richness (**a**), Shannon diversity (**b**), and abundance (**c**) of ten sites along the latitudinal gradient. Diversity metrics were compared with Wilcoxon test based on the sampling nights of each site. Different letters show significant differences between sites.

**Figure 3:**
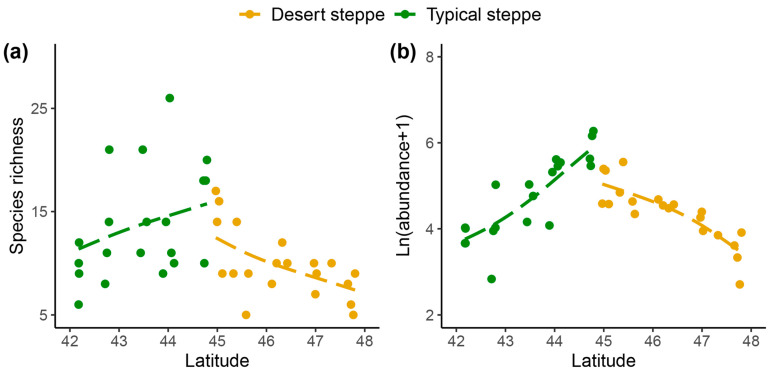
Patterns of (**a**) insect species richness and (**b**) abundance along latitude gradient in EEST. Patterns were analyzed with generalized additive models [Gaussian family, basis dimension (k) = 4].

**Figure 4:**
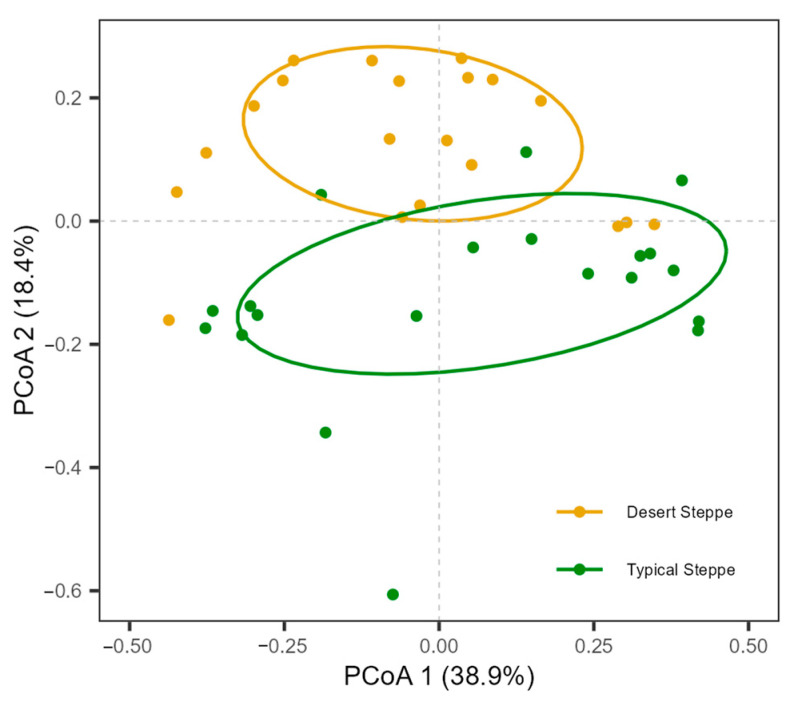
Principal coordinate analysis (PCoA) of insect communities across different grasslands. PCoA was generated using the Bray–Curtis dissimilarity method.

A total of 5244 individual insects were collected. These belonged to 99 species from 51 families and 7 orders, namely, 6 Orthoptera, 9 Hemiptera, 15 Diptera, 9 Coleoptera, 10 Hymenoptera, 1 Lepidoptera, and 1 Neuroptera (Table S2). The families with the highest abundance were Miridae (54.5% of species), Sarcophagidae (13.9%), and Cicadellidae (11.1%) (Figure S2). The most frequently collected species was *Rubrocuneocoris maculosus* with 2168 individuals; this species also showed the most extensive distribution, being present in all sites. Fifty-two species (53%) were found at one site (Figure S3). Overall, insect species richness (Figure 2a), species diversity (Figure 2b), and abundance (Figure 2c) of the typical steppe sites (1–5) were significantly higher (*p* < 0.005) than those of the desert steppe sites (6–10). Among ten sites, species abundance and diversity at Site 5 (a typical steppe) was higher than in all other sites. The most species-rich site was Site 5, and the most species-poor site was Site 10. The species richness increased significantly with latitude in the typical steppe, while decreasing significantly in the desert steppe (df = 2.7; F = 5.7; *p* < 0.01; Figure 3). The insect abundance showed an n-shaped distribution along the latitudinal gradient, with the greatest abundance in the typical steppe (df = 6; F = 3.814; *p* = 0.085; Figure 3) with linear increases (df = 1; F = 45.61; *p* < 0.001; Figure 3) but with linear declines in the desert steppe (df = 1.56; F = 27.32; *p* < 0.001; Figure 3). We found significant differences in insect communities across the different steppes, shown by the Bray–Curtis dissimilarity (PERMANOVA, pseudo-F = 4.7149, *p* < 0.001; Figure 4). 

### 3.2. The Effect of Environmental Variables on Insect Diversity

**Figure 5:**
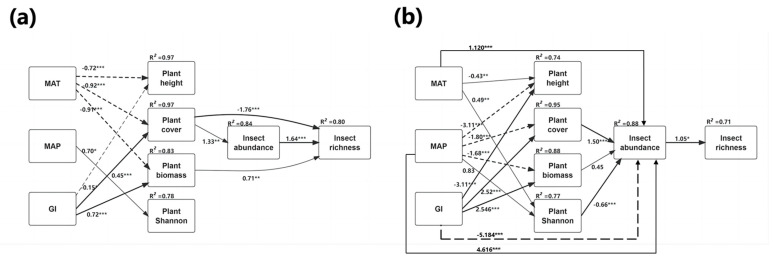
Path diagram for the structural equation model (SEM) for environmental factors on insect abundance and species richness in (**a**) typical steppe and (**b**) desert steppe. Statistically significant positive paths are indicated by solid arrows. Statistically significant negative paths are indicated by dashed arrows. The R^2^ values in each box indicate the amount of variation in that variable explained by the input arrows. Numbers next to arrows are unstandardized slopes. *p*-values significance level is < 0.05. * *p* ≤ 0.01, ** *p* ≤ 0.001, and *** *p* ≤ 0.0001.

The Mantel tests suggested that insect abundance was not strongly associated with either climate or plant attributes, although abundance was significantly and positively correlated with grazing intensity and geographical distance in the typical steppe (Table 2). In both the desert steppe and overall transect, except for precipitation and plant height, other environmental variables were all found to have significant effects on insect abundance; this was especially marked for temperature, grazing intensity, and plant biomass (Table 2). Consistent with the Mantel tests, the path analysis showed that climate and grazing intensity were significantly correlated with insect abundance across the different steppes (Figure 5), suggesting that both environmental variables and geographical distance influence insect communities and shape different diversity patterns in the different types of steppes. However, although the path analysis found an effect for precipitation, this was not corroborated by the Mantel tests across the whole transect (r = 0.06571; *p* = 0.1513; Table 2). This may reflect the lower sensitivity of the Spearman correlation method and the reduced effect of climatic background relative to the other factors. It should be noted that there was a relatively small average magnitude with large variations in the climate (Figure 5), suggesting that the corresponding climatic indicators may affect insect communities. These findings indicate the complexity of interactions between environmental factors on insect communities.

MAT and MAP had strong positive correlations with insect abundance, while GI had a negative effect on insect abundance in the desert steppe (Figure 5b). However, we found no significant direct effect of these environmental factors on insect diversity in the typical steppe (Figure 5a). It was also observed that climate and grazing intensity influence insect abundance indirectly through their effects on plants (Figure 5). The path analysis suggested that plant attributes, apart from the Shannon–Wiener index, showed strong negative correlations with temperature and precipitation across the different steppe, although there was a significant positive correlation with grazing intensity. However, there was no positive association between the Shannon–Wiener index and insect abundance. Instead, the index either had no effect or, in some cases, a negative effect on insects, while, of the plant attributes, plant cover showed a small but significant influence on insect abundance (typical steppe: standardized path coefficient = 1.33, *p* < 0.001; desert steppe: standardized path coefficient = 0.45, *p* < 0.05) and an overall positive influence on insect richness (Figure 5). Importantly, plant biomass also strongly affected the species richness in the typical steppe, but the effect was not mediated by insect abundance (standardized path coefficient = 0.71; *p* < 0.001) (Figure 5a), while insect abundance was able to effectively predict insect richness (typical: standardized path coefficient = 1.64, *p* < 0.001, Figure 5a; desert: standardized path coefficient = 1.05, *p* < 0.01, Figure 5b). However, no significant association between abundance and plant height was detected by SEM and Mantel tests (Table 2 and Figure 5).

### 3.3. Response of Different Orders and Species of Insects to Plant Attributes

**Figure 6:**
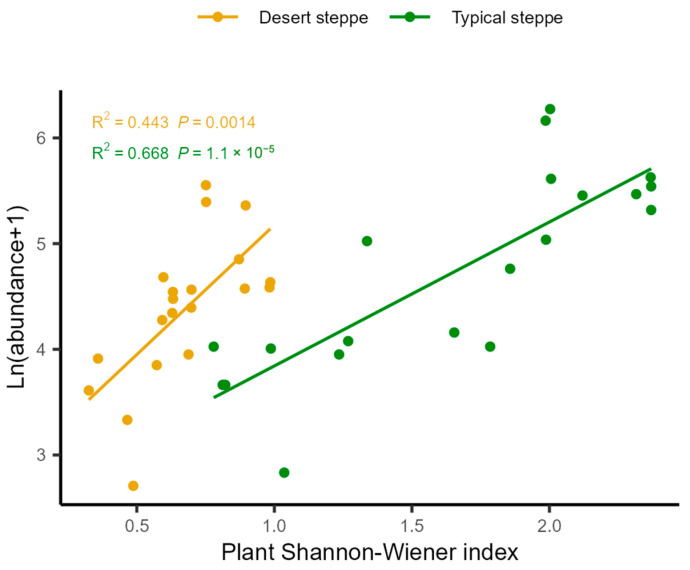
Relationships between plant Shannon–Wiener index and total insect abundance across different steppes.

**Figure 7:**
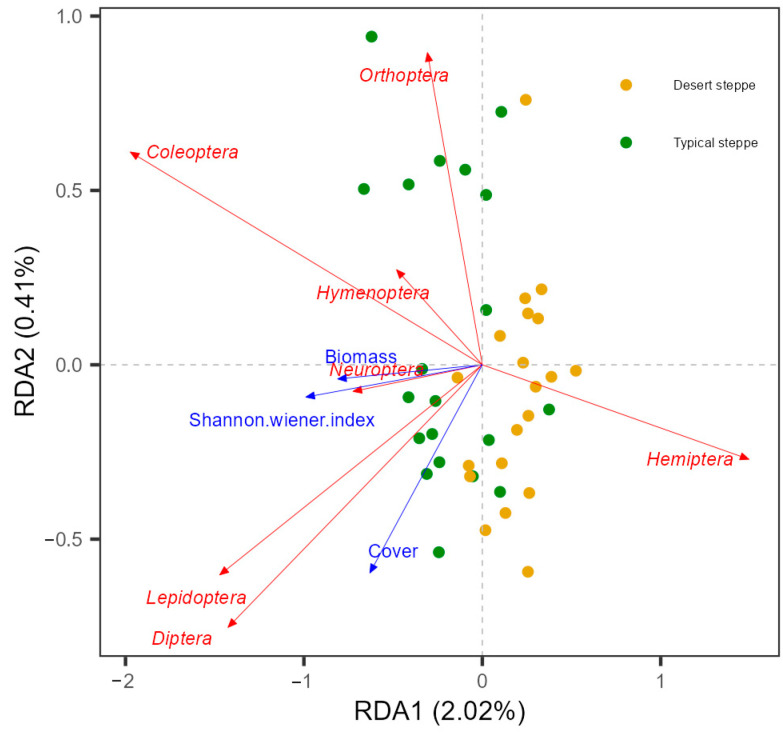
Insect taxonomic composition (orders) in relation to vegetation variables based on redundancy analysis (RDA). Significant vegetation variables are shown (inflation factor < 20, *p* < 0.05).

**Figure 8:**
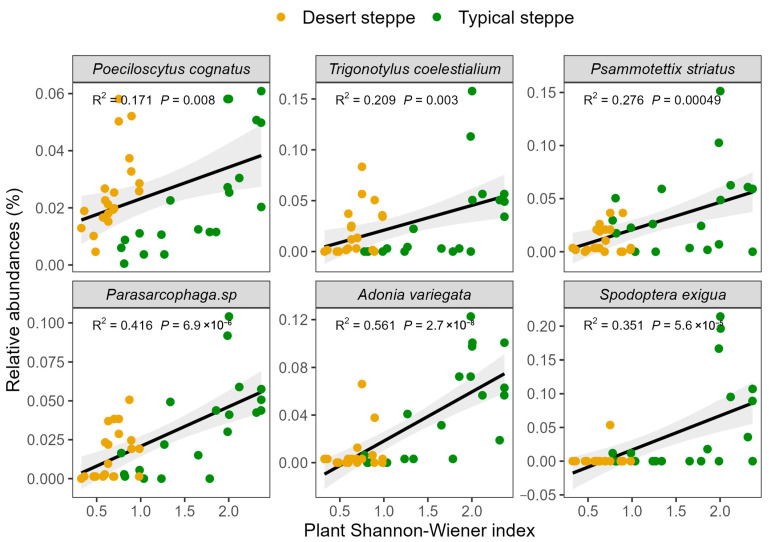
The relationships between the abundance of each insect species and plant Shannon–Wiener index in grazing treatments with the three plant diversity levels.

Analysis of the associations between insect abundance and plant characteristics showed that there was a positive relationship between total abundance and the Shannon–Wiener index (Figure 6). These relationships were quantified by RDA, examining the influence of plant characteristics on the relative abundances of the various insect orders (Figure 7). This showed that most of the variation in abundance was explained by axis 1, which was negatively associated with cover (R^2^ = 0.69, F = 12.451, *p* = 0.001), the Shannon–Wiener index (R^2^ = 0.496, F = 26.457, *p* < 0.0001), and biomass (R^2^ = 0.149, F = 5.941, *p* = 0.02). The second axis of the RDA was observed to be negatively associated with plant cover (R^2^ = 0.415, F = 24.099, *p* < 0.0001), height (R^2^ = 0.322, F = 16.121, *p* < 0.0001), and biomass (R^2^ = 0.195, F = 8.241, *p* = 0.007). The overall plant characteristics explained 2.56% of the variation in insect order abundance (F = 5.68, df = 3, *p* < 0.001). The orders represented in the insect communities were observed to group according to steppe type along the second RDA component. The strongest correlations between the insect community compositions were seen with the plant Shannon–Wiener index and biomass. In the typical steppes, the insect orders most closely associated with plant cover, biomass, and the Shannon–Wiener index were the Coleoptera, Diptera, and Lepidoptera, while in desert steppes, Hemiptera was strongly related to biomass and the Shannon–Wiener index. The six dominant insect species, namely, *Poeciloscytus cognatus*, *Trigonotylus coelestialium*, *Psammotettix striatus*, *Parasarcophaga* sp., *Adonia variegate*, and *Spodoptera exigua* were positively associated with the Shannon–Wiener index (Figure 8).

## 4. Discussion

In this study, we assessed the α and β diversity of insect communities and their associated environmental variables, observing that there were significant spatial differences in species diversity in different types of steppes (Figure 2). This is consistent with the recent findings of Enkhtur et al. [39] on moth distributions and diversities along latitudinal gradients in the temperate grasslands of Mongolia between 2018 and 2019. Here, we observed two distinct insect community structures along the latitudinal gradient, with the shift occurring at 44° N between Sites 5 and 6. We surmise that this change is the result of environmental factors and geographical distances, with the effects mediated by alterations in the vegetation. Greater levels of species richness and abundance were seen in the typical steppe sites compared with those in the desert steppe. However, the most abundant species *Poeciloscytus cognatus* and *Trigonotylus coelestialium* were less abundant in the typical steppe, possibly due to competitive exclusion. Plant heterogeneity was greater in the typical steppe, which might decrease insect competitive exclusion. This would allow the presence of multiple species or high richness at comparable proportions, specifically, similar abundance among species/high evenness. In contrast, the desert steppes showed reduced richness together with an increased abundance of insect species that were adapted to specific plants in these environments.

In the desert steppe, temperature had a positive direct effect on insect abundance (Figure 5b), possibly because these habitats are at a higher latitude, and temperatures are low. Thus, higher temperature will stimulate activity, especially in predatory species, as they feed more at higher temperatures [40]. We found no direct significant effect in the typical steppe (Figure 5a), possibly because the communities of this habitat are mainly composed of species adapted to relatively humid conditions. Meanwhile, our results showed that temperature had a positive effect on plant height and plant diversity in the desert steppe (Figure 5b). This suggests that, in addition to the direct effect, the influence of temperature on insect communities was indirectly mediated through alterations in plant height and plant diversity in the desert steppe, as plant diversity is often used as a proxy for food resource diversity for insects [22,23]. In contrast, temperature was significantly negatively correlated with plant attributes in the typical steppe, as has been previously observed [41], especially in arid or semi-arid environments [42], where water is restricted, and increased temperatures can not only reduce photosynthesis [43] but also increase evaporation, leading to further water stress [44], and thus reduce the plant biomass, height, and cover. 

As regards temperature, precipitation had a positive direct effect on insect abundance in the desert steppe; in the more humid environment, the typical steppe, there is no direct influence because precipitations are relatively abundant in these sampled sites. It is known that the amount of precipitation usually enhances the above-ground vegetation diversity in a temperate steppe [45,46], consistent with the results of the present study. For example, precipitation had a positive and significant correlation with the plant Shannon–Wiener index in the different steppes (Figure 4). Furthermore, both plant cover and biomass were greater in sites with higher precipitation (e.g., Sites 4 and 5) than in drier regions where the water in the soil may be insufficient for extensive plant growth. As vegetation provides insects with food (directly for the herbivorous species and indirectly for the predatory ones) and shelter sites, more vegetation biomass may allow insects to reach high species richness and abundance [47]. Overall, our findings indicate that the influence of climate factors on insect communities is complex in different steppes and varies according to vegetation characteristics and local climate conditions.

Our findings also showed the significant influence of grazing intensity on the structures of insect communities. This was shown by both the Mantel test (Table 2) and path analysis (Figure 5). We found a negative direct effect of grazing on insect abundance in the desert steppe, but no significant direct effect on insect diversity in the typical steppe. This is possibly because, in harsher, more arid habitats such as a desert steppe, excessive disturbance is more likely to have a negative impact on insect diversity. Due to the limited food resources of these habitats, large herbivores affect insect diversity by directly disturbing and dispersing their food resources and shortening their food chains [45]. Large herbivores can modify the vegetation substantially, thus affecting insect communities, particularly in terms of richness [48], complexity [49,50], and productivity [51]. The effects of grazing on insect communities are thus dependent on the degree to which grazing influences the vegetation characteristics. The present study found that grazing was positively correlated with plant cover and biomass in the different grasslands in correspondence with insect abundance (Figure 5), which was positively associated with the plant Shannon–Wiener index (Figure 6). This finding was supported by the RDA results (Figure 7). There is a close association between both plant cover and biomass with grassland productivity [52]. In terms of the bottom-up paradigm, the producers ultimately determine the amount of carbon entering the food web and, therefore, changes at this level, measured by altered plant diversity or biomass, will affect organisms in the upper trophic levels of the ecosystem [53,54]. A previous study observed that systems characterized by many or more productive plant species can support greater numbers of insects due to increases in the food supply [55]. We found that higher levels of plant cover and biomass were associated with greater insect abundance. Increases in plant biomass appeared to be associated largely with an altered abundance of dominant grass species, which would benefit grass-eating insects such as grasshoppers [56], confirming the influence of variables such as climate and grazing on plant biomass and hence on the insect communities. These factors also affected insect species richness (Figure 4) in the different steppes. However, we did not find that plant height influenced insect abundance in either steppe type, as has been previously reported [50,57]. 

The insect orders varied in their responses to plant characteristics, confirming previous reports where reduced biomass was associated with increased numbers of Coleoptera and Hemiptera and reduced numbers of Lepidoptera [58] and reductions in Hymenoptera and Lepidoptera [59]. Here, the RDA analysis showed that Orthoptera, Coleoptera, and Hymenoptera were likely to be influenced by altered plant biomass, and Neuroptera was associated with plant richness, while other orders, such as the Diptera and Lepidoptera, appeared to respond largely to plant structural features (Figure 7). As most Orthoptera are herbivorous, the amount of food available is likely to be necessary for their development [60], suggesting that, in this case, biomass might be more significant than the Shannon–Wiener index. In contrast, Neuroptera was more strongly influenced by the Shannon–Wiener index as it requires food quality rather than quantity. Other insect orders may be more strongly influenced by plant community structures as they tend to have specific habitat requirements, such as for refugees or oviposition [61]. In terms of insect species, the abundance of the six dominant species was positively associated with the Shannon–Wiener index (Figure 8). The RDA results confirmed the previous finding of the influence of grazing on abundance, indicating that both Orthopteran and Hemipteran species were influenced by grazing through modifications in plant community structures (Figure 7). Thus, it is apparent that the insect orders respond differently to plant characteristics and suggests that consideration of the changes at the order and species levels should be used in assessments of insect diversity in grasslands subjected to grazing. 

Our findings indicate that the differences in insect community structures seen across the different steppe types depend strongly on the richness of the plant species in the particular ecosystems [62]. These differences depend on three principal factors, namely, climatic variables, grazing intensity, and the diversity of the vegetation. Compared with other sampling methods, such as suction sampling [63], our sampling methods had some limitations, which may have caused some insect taxa loss, and there was a lack of prolonged monitoring and comprehensive interpretation. The precise mechanisms responsible for insect community variations, thus, need further investigation and clarification. Our findings emphasize the necessity of evaluating different insect taxa, as well as their trophic levels, to acquire a comprehensive understanding of the effects of climate change and grazing on invertebrate communities.

## 5. Conclusions

The present findings highlight the importance of a large-scale investigation of the impacts of climate and grazing in different grassland ecosystems. It was found that both climate and grazing influenced insect communities through the mediation of plant diversity, strongly supporting the significance of bottom-up effects in these ecosystems, especially in relation to grazing and climate change. The relative contributions of specific plant characteristics varied according to the steppe types and insect functional groups, with typical steppe and herbivores responding more strongly to vegetation diversity. Therefore, the response of insect diversity and its ecological service function in the steppe ecosystem under the combined influence of various factors, such as different grassland management methods, vegetation composition adjustment, and climate change, still needs long-term monitoring and evaluation.

## Figures and Tables

**Table 1:** Environmental characteristics at the study sites.

Number	Site	Steppe Type	MAT (°C) ^1^	MAP (mm) ^2^	GI (Sheep ha^−1^) ^3^
1	Duolun, Inner Mongolia	Typical	1.84 (0.011)	367.88 (0.286)	136.82
2	Zhenglan Banner, Inner Mongolia	Typical	1.85 (0.013)	416.68 (0.437)	63.43
3	Dalinor, Inner Mongolia	Typical	1.61 (0.041)	416.74 (26.674)	32.04
4	Xilinhot, Inner Mongolia	Typical	1.21 (0.038)	458.84 (29.321)	63.71
5	Xilinhot, Inner Mongolia	Typical	0.55 (0.053)	486.52 (2.165)	53.72
6	Abaga Banner, Inner Mongolia	Desert	0.19 (0.020)	394.99 (0.568)	51.53
7	Abaga Banner, Inner Mongolia	Desert	0.13 (0.029)	388.93 (3.847)	40.06
8	Khaldzan, Sukhbaatar, Mongolia	Desert	0.97 (0.355)	204.31 (6.573)	14.71
9	Sukhbaatar, Sukhbaatar, Mongolia	Desert	−0.46 (0.278)	243.53 (4.063)	7.61
10	Bulgan, Dornod, Mongolia	Desert	−1.69 (0.222)	222.20 (1.620)	4.91

^1^ MAT (°C): mean annual temperature; ^2^ MAP(mm): mean annual precipitation; ^3^ GI: grazing intensity.

**Table 2:** Effects of different environmental factors in the Mantel test analysis for each of the two steppe types and the whole transect in EEST.

Effect	Typical Steppe		Desert Steppe		The Whole Transect	
	*r*	*p*	*r*	*p*	*r*	*p*
MAT ^1^	−0.01958	0.5236	0.343	<0.01 *	0.3655	0.0001 ***
MAP ^2^	0.1145	0.1228	0.0708	0.1386	0.06571	0.1513
GI ^3^	0.2951	0.0175.	0.2734	<0.01 *	0.3563	0.0001 ***
PH ^4^	0.0407	0.3529	0.03031	0.3846	-0.01758	0.5553
PC ^5^	0.02726	0.3426	0.3864	0.0001 ***	0.1319	0.0229
PB ^6^	0.004527	0.4652	0.3433	<0.01 *	0.2003	<0.001 **
PS ^7^	0.2369	<0.01 **	0.2565	0.0134	0.1457	0.0275
ENV ^8^	0.1809	0.0429	0.3099	<0.001 **	0.2772	<0.001 **
GEO ^9^	0.3195	<0.001 **	0.3649	<0.001 **	0.3953	0.0001 ***

The partial regression coefficients (*r*) and associated *p*-values of the final model are reported from permutation test (nperm = 9999) if its significance level is <0.05. * *p* ≤ 0.01, ** *p* ≤ 0.001, and *** *p* ≤ 0.0001. ^1^ MAT: mean annual temperature; ^2^ MAP: mean annual precipitation; ^3^ GI: grazing intensities; ^4^ PH: plant height; ^5^ PC: plant cover; ^6^ PB: plant biomass; ^7^ PS: plant Shannon–Wiener index; ^8^ ENV: environmental changes; ^9^ GEO: geographical distance.

## Data Availability

The data presented in this study are available upon request from the corresponding author.

## Supplementary Materials

The following supporting information can be downloaded at: https://www.mdpi.com/article/10.3390/insects14020191/s1, Figure S1: Sample coverage of insects from 10 sites in the EEST; Figure S2: Relative abundance of insects at the family level from 10 sites in the EEST; Figure S3: Distribution of the number of sites occupied by the 100 insect species collected from 10 sites in the EEST; Table S1: Sampling site information in terms of their grassland types; *Sobs* = observed insect species richness, SC = sample coverage. Latitude and longitude data correspond to the centroid at each site; Table S2: List of species collected in 40 samples from 10 sites across the latitude gradient in the EEST. The table is filled with species occurrences (i.e., number of one site in which a species was collected regardless of the number of individuals). Table S3: Environmental variables of 40 samples from 10 sites across the latitude gradient in the EEST.

## References

[1] Woodward S.L., Woodward S.L. (2008). The temperate grassland biome. Grassland Biomes.

[2] Jiao C.C., Yu G.R., Ge J.P., Chen X., Zhang C., He N.P., Chen Z., Hu Z.M. (2017). Analysis of spatial and temporal patterns of aboveground net primary productivity in the Eurasian steppe region from 1982 to 2013. Ecol. Evol..

[3] Hou X.Y. (2012). Discussion on setting up ecological transects in Eastern Eurasian temperate steppe. Chin. J. Grassl..

[4] Gao L., Hou X.Y., Wang Z., Han W.J., Yun X.J. (2019). Effects of heavy grazing on soil nitrogen mineralization and temperature sensitivity along the Eastern Eurasia Steppe Transect. Acta Ecol. Sin..

[5] Petermann J.S., Buzhdygan O.Y. (2021). Grassland biodiversity. Curr. Biol..

[6] Quigley T.P., Amdam G.V., Harwood G.H. (2019). Honey bees as bioindicators of changing global agricultural landscapes. Curr. Opin. Insect Sci..

[7] Gerlach J., Samways M., Pryke J. (2013). Terrestrial invertebrates as bioindicators: An overview of available taxonomic groups. J. Insect Conserv..

[8] Mendez-Rojas D.M.M., Cultid-Medina C., Escobar F. (2021). Influence of land use change on rove beetle diversity: A systematic review and global meta-analysis of a mega-diverse insect group. Ecol. Indic..

[9] Miao B.G., Peng Y.Q., Yang D.R., Kubota Y., Economo E.P., Liu C. (2021). Climate and land-use interactively shape butterfly diversity in tropical rainforest and savanna ecosystems of southwestern China. Insect Sci..

[10] Bale J.S., Masters G.J., Hodkinson I.D., Awmack C., Bezemer T.M., Brown V.K., Butterfield J., Buse A., Coulson J.C., Farrar J. (2002). Herbivory in global climate change research: Direct effects of rising temperature on insect herbivores. Glob. Chang. Biol..

[11] Brehm G., Süssenbach D., Fiedler K. (2003). Unique elevational diversity patterns of geometrid moths in an Andean montane rainforest. Ecography.

[12] Axmacher J.C., Brehm G., Hemp A., Tünte H., Lyaruu H.V.M., Müller-Hohenstein K., Fiedler K. (2009). Determinants of diversity in afrotropical herbivorous insects (Lepidoptera: Geometridae): Plant diversity, vegetation structure or abiotic factors?. J. Biogeogr..

[13] Sponseller R. (2010). Precipitation pulses and soil CO2 flux in a Sonoran Desert ecosystem. Glob. Chang. Biol..

[14] Zhu H., Wang D.L., Wang L., Bai Y.G., Fang J., Liu J. (2012). The effects of large herbivore grazing on meadow steppe plant and insect diversity. J. Appl. Ecol..

[15] Sangle P.M., Satpute S.B., Khan F.S., Rode N.S. (2015). Impact of climate change on insects. Trends Biosci..

[16] Zhu H., Wang D., Guo Q., Liu J., Wang L. (2015). Interactive effects of large herbivores and plant diversity on insect abundance in a meadow steppe in China. Agric. Ecosyst. Environ..

[17] Jerrentrup J.S., Wrage-Monnig N., Rover K.U., Isselstein J. (2014). Grazing intensity affects insect diversity via sward structure and heterogeneity in a long-term experiment. J. Appl. Ecol..

[18] Zhao Z., Reddy G.V., Wei S., Zhu M., Zhang K., Yu H., Wang Z., Jiang Q., Zhang R. (2018). Plant cover associated with aboveground net primary productivity (ANPP) mediates insect community composition in steppes of Northwest China. J. Asia Pac. Entomol..

[19] Poyry J., Lindgren S., Salminen J., Kuussaari M. (2004). Restoration of butterfly and moth communities in semi-natural grasslands by cattle grazing. Ecol. Appl..

[20] Jiang L., Wang S., Pang Z., Wang C., Kardol P., Zhou X., Rui Y., Lan Z.C., Wang Y., Xu X. (2016). Grazing modifies inorganic and organic nitrogen uptake by coexisting plant species in alpine grassland. Biol. Fertil. Soils.

[21] Zhu H., Wang W., Qu Y., Fu J., Jiang S., Wang D., Kaplan I., Ren B. (2018). Resource-mediated effects of grazing and irrigation on insect diversity in a meadow steppe. Insect Conserv. Divers..

[22] Ribas C.R., Schoereder J.H., Pic M., Soares S.M. (2003). Tree heterogeneity, resource availability, and larger scale processes regulating arboreal ant species richness. Austral Ecol..

[23] Vasconcelos H.L., Maravalhas J.B., Neves K.C., Pacheco R., Vieira J., Camarota F.C., Izzo T.J., Araújo G.M. (2019). Congruent spatial patterns of ant and tree diversity in neotropical savannas. Biodivers. Conserv..

[24] Zhao Y., Ding Y., Hou X.Y., Li F.Y., Han W.J., Yun X. (2017). Effects of temperature and grazing on soil organic carbon storage in grasslands along the Eurasian steppe eastern transect. PLoS ONE.

[25] Ren H.R., Zhou G.S., Zhang X.S. (2011). Estimation of green aboveground biomass of desert steppe in inner Mongolia based on red-edge reflectance curve area method. Biosyst. Eng..

[26] Schaffers A.P., Raemakers I.P., Sýkora K.V., Ter Braak C.J. (2008). Arthropod assemblages are best predicted by plant species composition. Ecology.

[27] Nonnaizab, Qi B.Y., Li Y.B. (1999). Insects of Inner Mongolia China.

[28] Cao R. (2017). Illustrations of Common Plants in Inner Mongolia.

[29] R Core Team (2022). R: A Language and Environment for Statistical Computing.

[30] Chao A., Chiu C.H., Hsieh T.C., Davis T., Nipperess D.A., Faith D.P. (2014). Rarefaction and extrapolation of phylogenetic diversity. Methods Ecol. Evol..

[31] Hsieh T.C., Ma K.H., Chao A. (2016). iNEXT: An R package for rarefaction and extrapolation of species diversity (Hill numbers). Methods Ecol. Evol..

[32] Oksanen J., Blanchet F.G., Kindt R., Legendre P., O’hara R.B., Simpson G.L., Solymos P., Stevens M.H., Wagner H. (2018). Vegan: Community Ecology Package.

[33] Mantel N. (1967). The detection of disease clustering and a generalized regression approach. Cancer Res..

[34] Legendre P., Fortin M.J. (1989). Spatial pattern and ecological analysis. Vegetatio.

[35] Shipley B. (2009). Confirmatory path analysis in a generalized multilevel context. Ecology.

[36] Laliberte E., Tylianakis J.M. (2012). Cascading effects of longterm land-use changes on plant traits and ecosystem functioning. Ecology.

[37] Sanchez G. (2013). PLS Path Modeling with R.

[38] Bates D., Maechler M., Bolker B., Walker S. (2014). LME4: Linear Mixed-Effects Models Using Eigen and S4. https://cran.r-project.org/web/packages/lme4/index.html.

[39] Enkhtur K., Brehm G., Boldgiv B., Pfeifer M. (2021). Alpha and beta diversity patterns of macro-moths reveal a breakpoint along a latitudinal gradient in Mongolia. Sci. Rep..

[40] Frank T., Bramböck M. (2016). Predatory beetles feed more pest beetles at rising temperature. BMC Ecol..

[41] De Boeck H.J., Lemmens C.M., Zavalloni C., Gielen B., Malchair S., Carnol M., Merckx R., Van den Berge J., Ceulemans R., Nijs I. (2008). Biomass production in experimental grasslands of different species richness during three years of climate warming. Biogeosciences.

[42] Ma W., Liu Z., Wang Z., Wang W., Liang C., Tang Y., He J.S., Fang J. (2010). Climate change alters interannual variation of grassland aboveground productivity: Evidence from a 22-year measurement series in the Inner Mongolian grassland. J. Plant Res..

[43] Xu Z.Z., Zhou G.S. (2005). Effects of water stress and high nocturnal temperature on photosynthesis and nitrogen level of a perennial grass Leymus chinensis. Plant Soil.

[44] De Boeck H.J., Lemmens C.M., Bossuyt H., Malchair S., Carnol M., Merckx R., Nijs I., Ceulemans R. (2006). How do climate warming and plant species richness affect water use in experimental grasslands?. Plant Soil.

[45] Yan H., Liang C., Li Z., Liu Z., Miao B., He C., Sheng L. (2015). Impact of Precipitation Patterns on Biomass and Species Richness of Annuals in a Dry Steppe. PLoS ONE..

[46] Yang Y., Dou Y., An S. (2017). Environmental driving factors affecting plant biomass in natural grassland in the Loess Plateau, China. Ecol. Indic..

[47] Tsafack N., Xie Y., Wang X., Fattorini S. (2019). Influence of Climate and Local Habitat Characteristics on Carabid Beetle Abundance and Diversity in Northern Chinese Steppes. Insects.

[48] Joern A. (2005). Disturbance by fire frequency and bison grazing modulate grasshopper assemblages in tallgrass prairie. Ecology.

[49] Pöyry J., Luota M., Paukkunen J., Pykälä J., Raatikainen K., Kuussaari M. (2006). Different responses of plants and herbivore insects to a gradient of vegetation height: An indicator of the vertebrate grazing intensity and successional age. Oikos.

[50] KÅrösi Á., Batáry P., Orosz Á., Rédei D., Báldi Á. (2012). Effects of grazing, vegetation structure and landscape complexity on grassland leafhoppers (Hemiptera: Auchenorrhyncha) and true bugs (Hemiptera: Heteroptera) in Hungary. Insect Conserv. Divers..

[51] Perner J., Wytrykush C., Kahmen A., Buchmann N., Egerer I., Creutzburg S., Odat N., Audorff V., Weisser W.W. (2005). Effects of plant diversity, plant productivity and habitat parameters on arthropod abundance in montane European grasslands. Ecography.

[52] Wang L., Liu C., Alves D.G., Frank D.A., Wang D.L. (2015). Plant diversity is associated with the amount and spatial structure of soil heterogeneity in meadow steppe of China. Landsc. Ecol..

[53] Haddad N.M., Tilman D., Haarstad J., Ritchie M., Knops J.M.H. (2001). Contrasting effects of plant richness and composition on insect communities: A field experiment. Am. Nat..

[54] Scherber C., Eisenhauer N., Weisser W.W., Schmid B., Voigt W., Fischer M., Schulze E.D., Roscher C., Weigelt A., Allan E. (2010). Bottom-up effects of plant diversity on multitrophic interactions in a biodiversity experiment. Nature.

[55] Haddad N.M., Crutsinger G.M., Gross K., Haarstad J., Knops J.M.H., Tilman D. (2009). Plant species loss decreases arthropod diversity and shifts trophic structure. Ecol. Lett..

[56] Specht J., Scherber C., Unsicker S.B., Köhler G., Weisser W.W. (2008). Diversity and beyond: Plant functional identity determines herbivore performance. J. Anim. Ecol..

[57] Siemann E. (1998). Experimental tests of effects of plant productivity and diversity on grassland arthropod diversity. Ecology.

[58] Dennis P., Skartveit J., McCracken D.I., Pakeman R.J., Beaton K., Kunaver A., Evans D.M. (2008). The effects of livestock grazing on foliar arthropods associated with bird diet in upland grasslands of Scotland. J. Appl. Ecol..

[59] Sjödin N.E., Bengtsson J., Ekbom B. (2008). The influence of grazing intensity and landscape composition on the diversity and abundance of flower-visiting insects. J. Appl. Ecol..

[60] Berner D., Blanckenhorn W.U., Körner C. (2005). Grasshopper cope with low host plant quality by compensatory feeding and food selection: N limitation challenged. Oikos.

[61] Morris M.G. (2000). The effects of structure and its dynamics on the ecology and conservation of arthropods in British grasslands. Biol. Conserv..

[62] Wang L., Wang D.L., Liu J.S., Huang Y., Hodgkinson K.C. (2011). Diet selection variation of a large herbivore in a feeding experiment with increasing species numbers and different plant functional group combinations. Acta Oecol..

[63] Kruess A., Tscharntke T. (2002). Contrasting responses of plant and insect diversity to variation in grazing intensity. Biol. Conserv..

