# Supplementary figures and images for: Patterns and Drivers of Aboveground Insect Diversity along Ecological Transect in Temperate Grazed Steppes of Eastern Eurasian
Source: Insects. 2023 Feb 15;14(2):191. doi: 10.3390/insects14020191 (PMC9964858; doi:10.3390/insects14020191)

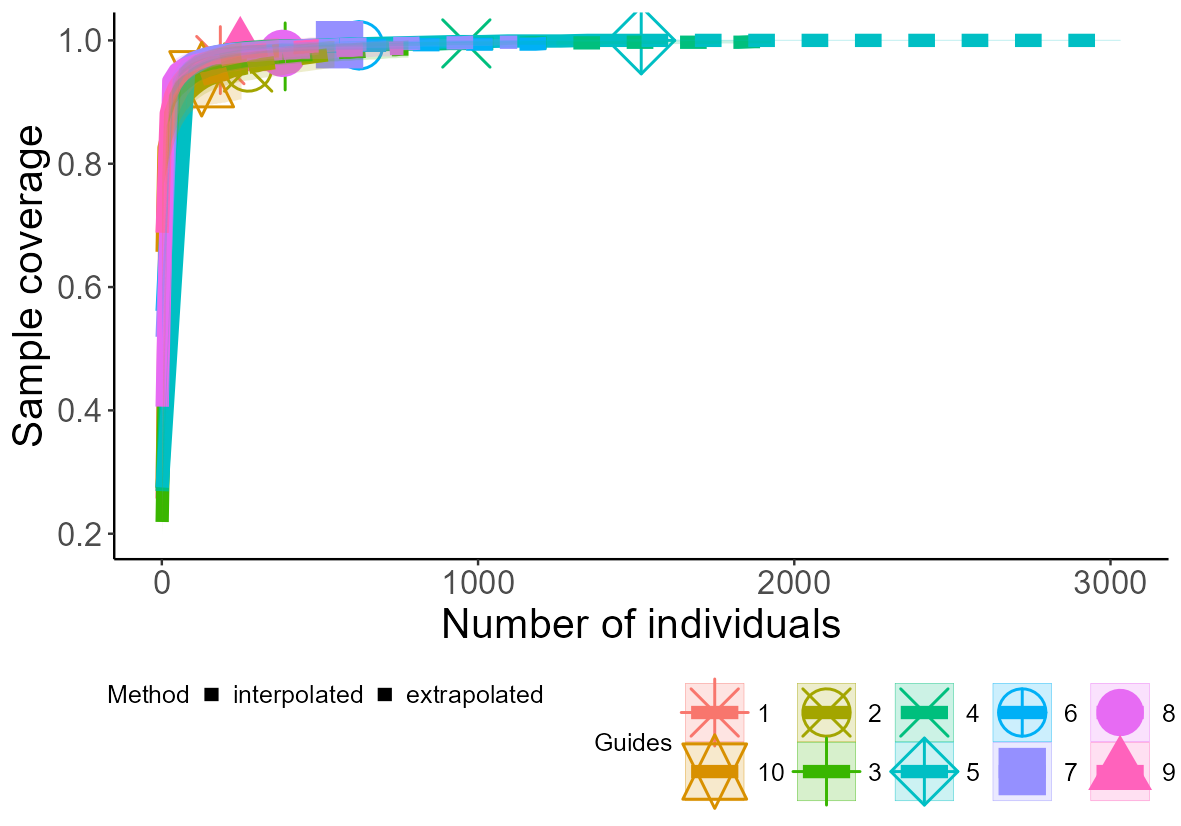

Supplement: Supplementary file 1 [file insects-14-00191-s001.zip › Supplementary Materials Figure S1.tiff]

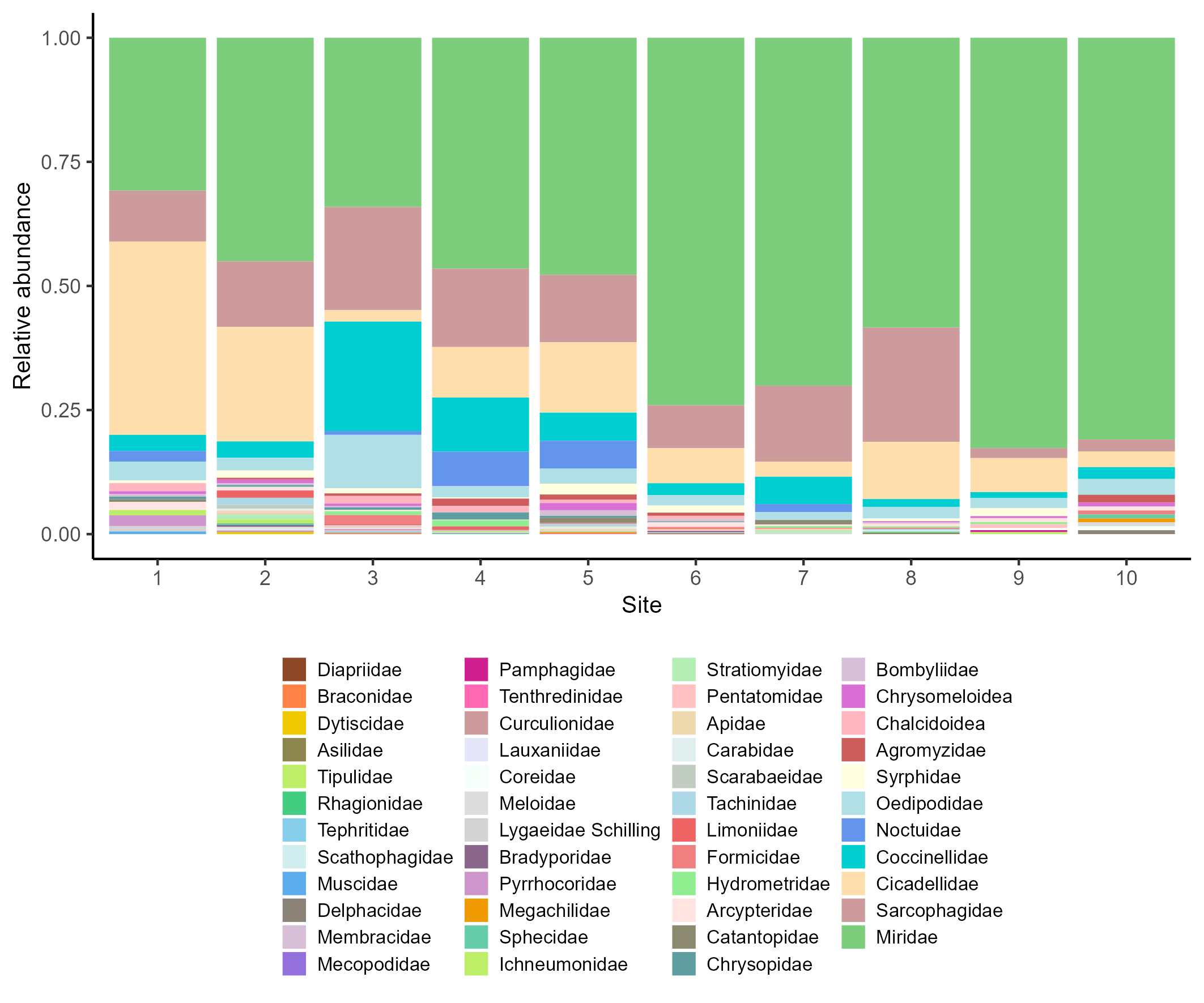

Supplement: Supplementary file 1 [file insects-14-00191-s001.zip › Supplementary Materials Figure S2.tiff]

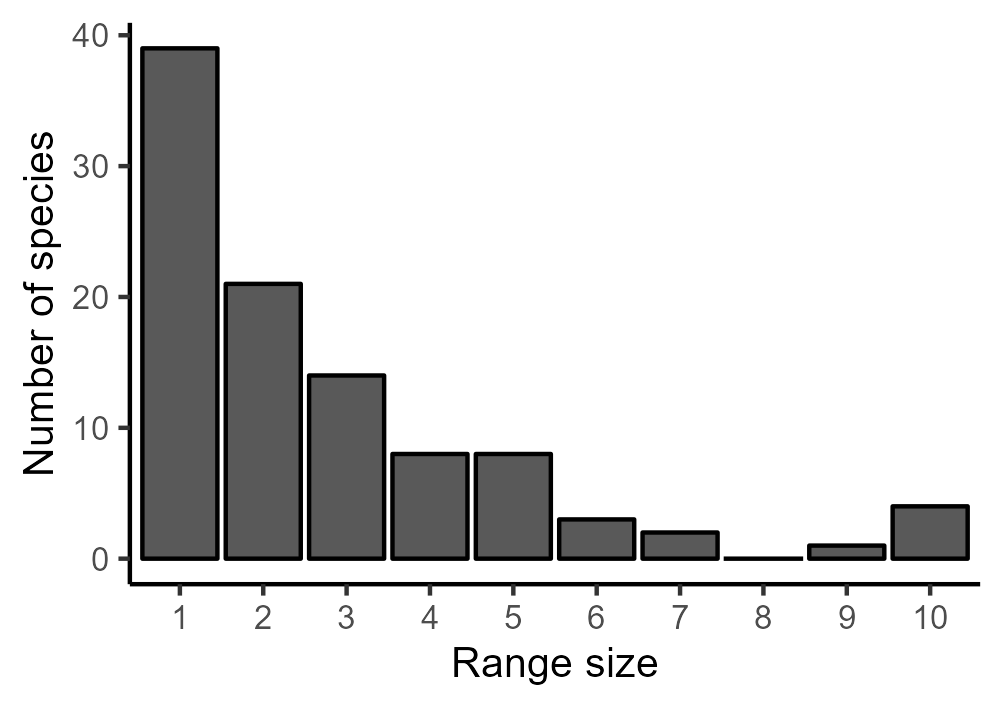

Supplement: Supplementary file 1 [file insects-14-00191-s001.zip › Supplementary Materials Figure S3.tiff]
